# 

**Published:** 1903-06-15

**Authors:** 


### ANNOUNCEMENTS.

At the annual meeting of the Chicago Dental Society Dr. D. M. Gallie was elected President and A. E. Morey, Corresponding Secretary.

---

Jackson (Mich.) Dental Society held its annual meeting April 20, 1903, and elected the following officers: Pres., J. W. Lyon; Secy. and Treas., R. O. Curtis.

---

Muskegon (Mich.) Dental Association held its annual meeting April 28, and elected the following officers: Pres., W. H. Miller; Secy. and Treas., J. G. Benjamin.

---

Dawson, Ky., has passed ordinance compelling dentists and physicians to pay a license tax of $100 a year, and Whittier, Cal., has taxed each physician and dentist $6 per year.

---

Dr. Wm. E. Walker, Dean of the Faculty, Herman B. Gessner, Jules J. Sarazin and Otto Lerch, all holding prominent chairs in the New Orleans Dental College, have resigned.

---

Columbus (O.) Dental Society held its annual meeting and banquet April 28, 1903, and elected the following officers:

Pres., H. M. Semans; Vice-pres., E. C. Sherman; Secy., H. Cooper; Treas., E. M. Fisher.

—✦—

PEORIA (ILLS.) DENTAL SOCIETY held its annual meeting and banquet May 2, 1903, and elected the following officers: Pres., J. D. Nicol; Vice-pres., Geo. T. Gray; Secy., R. L. Graham; Treas., W. A. Johnston.

—✦—

THE Illinois State Dental Society met at Bloomington, May 12 to 14. Dr. F. H. McIntosh, of Bloomington, was elected President and Dr. H. J. Goslee, of Chicago, was elected Secretary. The next meeting will be held at Peoria, May 10, 1904.

—✦—

DETROIT (MICH.) DENTAL SOCIETY held its annual meeting May 14, 1903, and elected the following officers: Pres., G. B. Watkins; Vice-pres., C. P. Wood; Secy., Wm. A. Griffin; Treas., G. C. Bowles; Member Board of Censors, M. L. Hogarth.

—✦—

DR. B. L. THORPE, of St. Louis, was elected President of the Missouri State Board of Dental Examiners, and Dr. C. A. Rubey, of Clinton, was elected Secretary, at a recent meeting of this Board. Seven candidates were examined and only three were licensed to practice.

—✦—

CENTRAL MICHIGAN DENTAL ASSOCIATION held its annual meeting at Grand Ledge, May 14, 1903, and elected the following officers: Pres., J. H. Armstrong; Vice-pres., P. F. Hines; Secy., G. T. Smith; Treas., S. A. Horning; Ex. Com., C. E. Whitmore, J. J. Green, J. P. Taylor.

—✦—

EASTERN INDIANA DENTAL ASSOCIATION held its annual meeting at Elwood, May, 14, 1903, and elected the following officers: Pres., F. S. Anderson; Vice-pres., W. B. Binfield; Sec'y and Treas., H. F. Hussey; The next meeting will be held at Richmond the second Tuesday in May, 1904.

—✦—

THE National Association of Dental Faculties will convene in the ball-room of the Battery Park Hotel, Asheville, N. C., at 11 a.m., July 24, 1903. The executive committee will meet at the same time and place, Thursday, July 23, at 2:30 p.m. Any one having business with this committee is hereby notified to be present at that time.

—✦—

THE National Dental Association meets in Asheville, N. C., Tuesday, July 28, 1903.

Preparations are being made for one of the best meetings in the history of the Association. The section officers are preparing a program which, from a scientific and practical standpoint, will be difficult to excel. The clinics will be made a special feature.

—✦—

All dentists interested in the advancement of the profession should attend this meeting.

All State and local societies should elect delegates who will be sure to attend the National meeting, they being entitled to one delegate for every six of their members.

The usual railroad rates will be had on all roads in the United States and part of Canada—one fare and a third, on the certificate plan. A. H. PECK, Rec. Sec'y.

—✦—

THE Michigan State Dental Society will meet at Petoskey, a delightful and popular summer resort, July 6, 7, 8 and 9, and it is expected that a large attendance and a fine program will make this one of the best meetings ever held by this society. A fine literary program has been prepared, and a large number of clinics are promised. The meeting will be held at the Arlington Hotel, a very commodious and well-kept hotel. Special rates are promised, and every one will be well taken care of. Bring your wife along and make this your summer outing, as many dentists are planning to do. Buy round-trip summer tourist tickets, as these give low rates and all kinds of stop-over privileges. Boats from Chicago and Cleveland make a pleasant way of getting to Petoskey.

—✦—

THE Toledo Dental Society entertained the Washtenaw Dental Society Friday, May 8, in a most delightful way. A large part of the day was spent in visiting the various industrial enterprises of the city. At five o'clock the venerable and highly-esteemed Dr. C. H. Harroun entertained the society with a paper on personal reminiscences. after which a sumptuous banquet was served in the large dining-room of the famous Boody House. The delegation was then escorted to the theater to enjoy a "Night Out." When we arrived home the next day we had a feeling that there were lots of good people in this world, and life was really worth living. The best of it is that the Toledo dentists didn't seem to think they were doing enough for their guests. Just how much more they are capable of doing we don't know, but we thought about as much good fellowship was crowded into those few hours as they were capable of holding.

